# Supplementary material for: Characterization of Charge States in Conducting Organic Nanoparticles by X-ray Photoemission Spectroscopy
Source: Materials (Basel). 2021 Apr 19;14(8):2058. doi: 10.3390/ma14082058 (PMC8072544; doi:10.3390/ma14082058)
Supplement: Supplementary file 1 [file materials-14-02058-s001.zip › materials-1167236-supplementary.pdf]

Supplementary Material

Characterization of Charge States in Conducting Organic Nanoparticles by X-Ray Photoemission Spectroscopy

Jordi Fraxedas [1,*], Antje Vollmer [2], Norbert Koch [3], Dominique de Caro [4,*] Kane Jacob [4], Christophe Faulmann [4] and Lydie Valade [4]

[1] Catalan Institute of Nanoscience and Nanotechnology (ICN2), CSIC and BIST, Campus UAB, Bellaterra, 08193 Barcelona, Spain
[2] Helmholtz Zentrum Berlin Materialien & Energie GmbH BESSY, D-12489 Berlin, Germany; antje.vollmer@helmholtz-berlin.de
[3] Institute of Physics, Humboldt University, D-12489 Berlin, Germany; norbert.koch@physik.hu-berlin.de
[4] LCC-CNRS, Université de Toulouse, CNRS, UPS, F-31077 Toulouse, France; kane.jacob@lcc-toulouse.fr (K.J.); christophe.faulmann@cemes.fr (C.F.); lydie.valade@lcc-toulouse.fr (L.V.)
* Correspondence: jordi.fraxedas@icn2.cat (J.F.); dominique.decaro@lcc-toulouse.fr (D.d.C.)

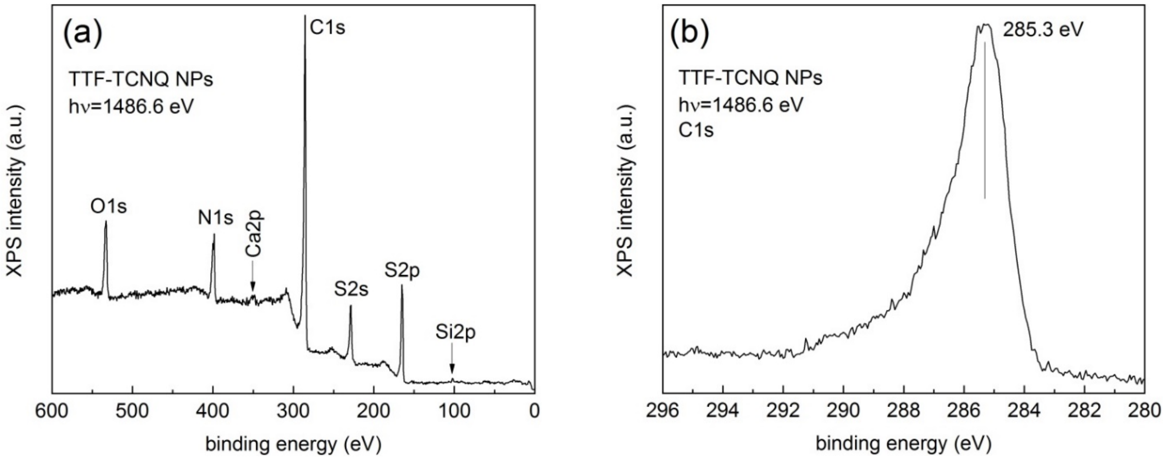

Figure S1. XPS survey (**a**) and C1s (**b**) spectra of a dispersion of NPs of TTF-TCNQ stabilized with *n*-octylamine measured with monochromatic 1486.6 eV photons.

In (a) the main lines are identified together with the contribution from traces of Si and Ca (arrows). The estimated at. % is: 71.3 [C], 12.8 [S], 8.2 [N], 6.5 [O], 0.5 [Si], 0.7 [Ca]. The nominal 1:1 stoichiometry of TTF-TCNQ is $C_{18}H_8S_4N_4$ (TTF=$C_6H_4S_4$ and TCNQ=$C_{12}H_4N_4$), so that [S]/[N]=1 and [C]/[S]=[C]/[N]=4.5. From the estimated at.% we obtain [C]/[S]=5.6, [C]/[N]=8.7 and [S]/[N]=1.6. The excess of C arises from the remaining n-octylamine stabilizer and from contamination due to the exposure to the atmosphere while the [S]/[N] > 1 ratio indicates an excess of TTF. The main contribution to the C1s line in (b) is centred at 285.3 eV, which arises from C-C and C-H bonding. The shoulder at higher binding energies contains contributions from C-S, C-N and C-O bonding.

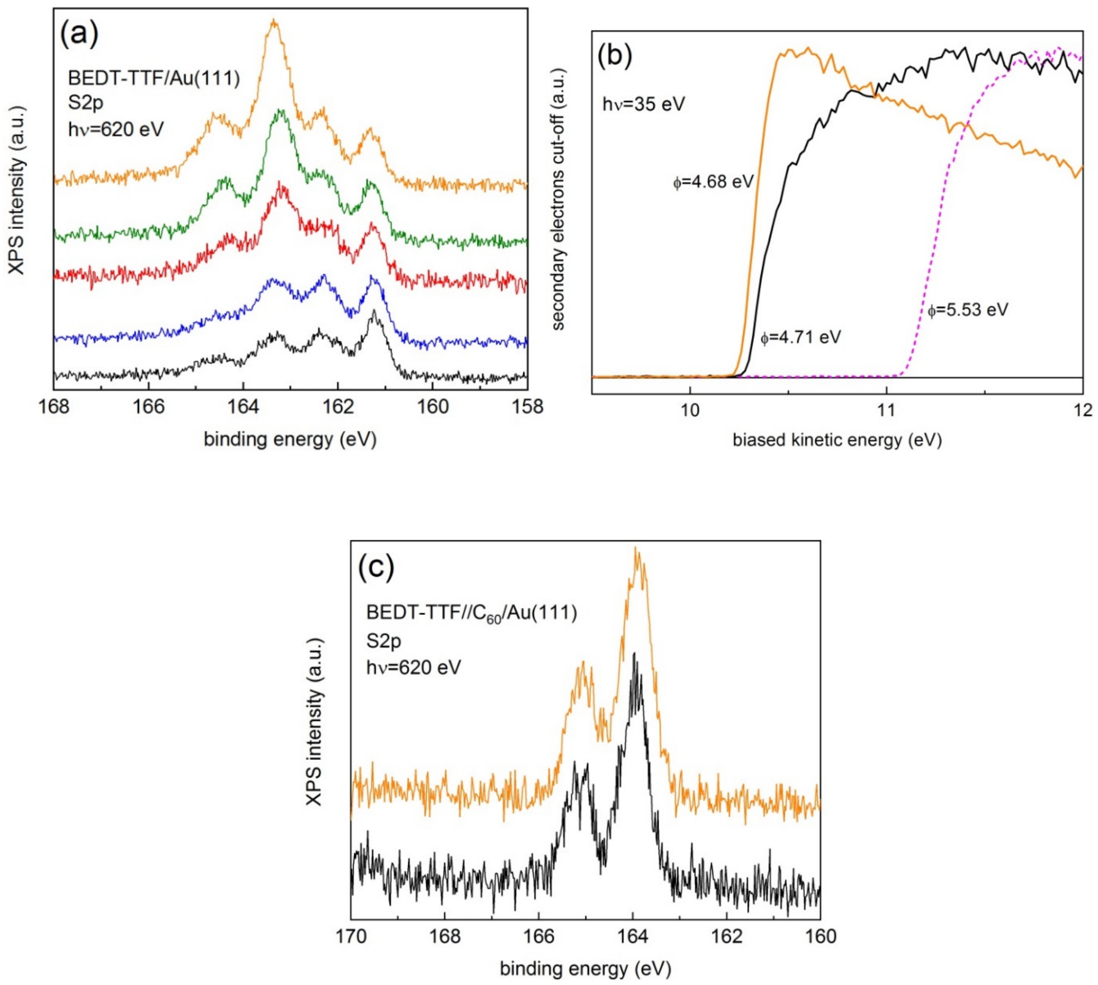

Figure S2. (**a**) XPS S2p lines of BEDT-TTF/Au(111) as a function of coverage measured with 620 eV photons, (**b**) work functions and (**c**) XPS S2p lines determined for thin BEDT-TTF films on C60-covered Au(111) substrates determined with 35 and 620 eV photons, respectively.

(a) High-resolution XPS S2p lines of BEDT-TTF/Au(111) as a function of coverage obtained with 620 eV photons. Colour codes: 0.08 ML (black line), 0.5 ML (blue line), 1 ML (red line), 6 ML (green line) and a thick BEDT-TTF film (orange line). (b) Photoemission onset determined with 35 eV photons for a clean Au(111) substrate (discontinuous magenta line), and for 0.17 and 16 ML BEDT-TTF films (continuous black and orange lines, respectively) grown on a 5 nm thick C60 film on Au(111) showing the corresponding work functions. (c) High-resolution XPS S2p lines of BEDT-TTF films grown on a 5 nm thick C_{60} film on Au(111) as a function of coverage obtained with 620 eV photons. See colour codes in (b) for the corresponding coverages.

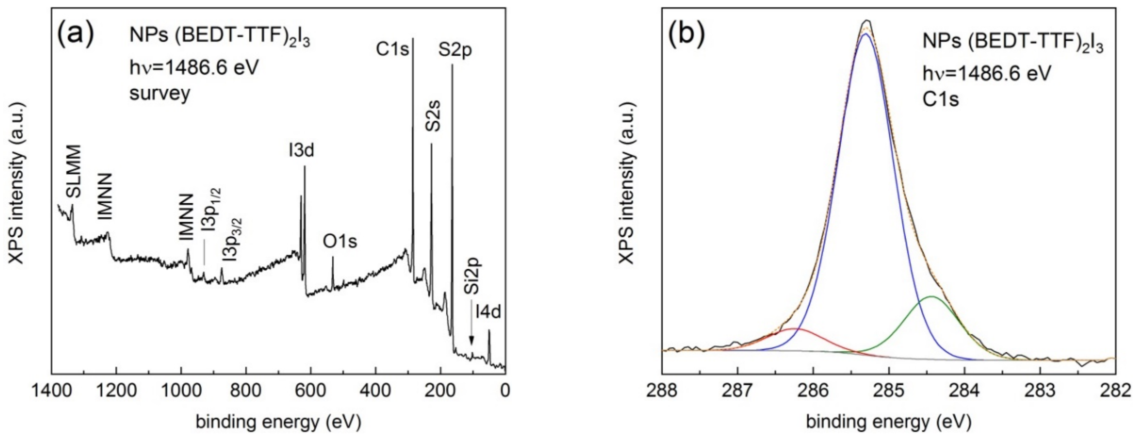

Figure S3. XPS survey (**a**) and C1s (**b**) spectra of a dispersion of NPs of $(BEDT\text{-}TTF)_2I_3$ measured with monochromatic 1486.6 eV photons.

In (a) the main lines are identified together with the contribution from traces of Si (arrow). The main contribution to the C1s line in (b) is centred at 285.3 eV, which arises from C-C and C-H bonding. The shoulder at higher binding energies (286.2 eV) contains contributions from C-S bonding. The feature centred at 284.4 eV most probably arises from the conductive carbon tape strips used to fix the nanoparticles on the sample holder.
